# Antimicrobial use before and during COVID-19: data from 108 Veterans Affairs medical centers

**DOI:** 10.1017/ash.2024.352

**Published:** 2024-08-07

**Authors:** Matthew Bidwell Goetz, Tina Willson, Michael A. Rubin, Vanessa W. Stevens, Christopher J. Graber

**Affiliations:** 1 VA Greater Los Angeles Healthcare System and David Geffen School of Medicine at UCLA, Los Angeles, CA, USA; 2 VA Salt Lake Informatics, Decision-Enhancement, and Analytic Sciences (IDEAS) Center, VA Salt Lake City Health Care System and University of Utah School of Medicine, Salt Lake City, UT, USA

## Abstract

**Objective:**

Inpatient antibiotic use increased during the early phases of the COVID-19 pandemic. We sought to determine whether these changes persisted in persons with and without COVID-19 infection.

**Design:**

Retrospective cohort analysis.

**Setting:**

108 Veterans Affairs (VA) facilities.

**Patients:**

Persons receiving acute inpatient care from January 2016 to October 2022.

**Methods:**

Data on antibacterial use, patient days present, and COVID-19 care were extracted from the VA Corporate Data Warehouse. Days of therapy (DOT) per 1000 days present (DP) were calculated and stratified by Centers for Disease Control and Prevention-defined antibiotic classes.

**Results:**

Antibiotic use increased from 534 DOT/1000 DP in 11/2019–2/2020 to 588 DOT/1000 DP in 3/2020–4/2020. Subsequently, antibiotic use decreased such that total DOT/1000 DP was 2% less in 2020 as a whole than in 2019. Driven by treatment for community acquired pneumonia, antibiotic use was 30% higher in persons with COVID-19 than in uninfected persons in 3/2020–4/2020, but only 4% higher for the remainder of 2020. In 2022 system-wide antibiotic use was 9% less in persons with COVID-19; however, antibiotic use remained higher in persons with COVID-19 in 25% of facilities.

**Discussion:**

Although antibiotic use increased during the early phases of the COVID-19 pandemic, overall use subsequently decreased to below previous baseline levels and, in 2022, was less in persons with COVID-19 than in persons without COVID-19. However, further work needs to be done to address variances across facilities and to determine whether current levels of antibiotic use in persons with COVID-19 are justified.

## Summary

During the first two months of the COVID-19 pandemic at 108 VA hospitals, antibiotic use in infected persons was 30% higher than in uninfected persons; use quickly decreased and was 9% less than in uninfected persons in 2022.

## Introduction

Prompted by concerns about potential bacterial coinfection and preliminary reports of the benefit of azithromycin in the treatment of COVID-19, during the early months of the COVID-19 pandemic 65–80% of patients with confirmed or suspected COVID-19 received antibiotic therapy.^1–5^ Following data demonstrating the lack of effectiveness of azithromycin and the low prevalence of documented community onset and secondary bacterial coinfection among persons with COVID-19,^6^ the rate of antibiotic administration among persons with COVID-19 diminished in the latter part of 2020, a trend that has continued.^2,7,8^ However, no large-scale studies have compared the long-term patterns of antibiotic use in persons with and without COVID-19. In this paper, we compare inpatient antibiotic use among hospitalized persons with and without COVID-19 as clinical care evolved from March 2020 to October 2022 within Veterans Affairs (VA) healthcare facilities.

## Methods

Data on antibacterial use, patient days present, and COVID-19 care for acute care units defined as inpatient admission, observation, intensive care and acute care medical or surgical units in 108 VA facilities were extracted from the Corporate Data Warehouse through the VA Informatics and Computing Infrastructure; facilities which provide limited acute inpatient services (e.g., VA complexity level three facilities), were excluded.^9^ Days of therapy (DOT) per 1,000 days present (DP) were calculated and stratified by Centers for Disease Control and Prevention (CDC) defined standardized antimicrobial administration ratio (SAAR) antibiotic classes (Supplementary Table 1).^10^

COVID-19 diagnoses were identified using the VA COVID-19 Shared Data Resource. This resource contains information on all veterans with a confirmed laboratory diagnosis of SARS-CoV-2 infection within the VA and those who were tested outside the VA with a VA clinical note confirming the diagnosis. The VA COVID-19 Shared Data Resource contains extensive demographic, clinical, pharmacologic, laboratory, vital sign, and clinical outcome information derived from multiple validated sources, including the VA Corporate Data Warehouse and the VA electronic medical record. The analysis plan for this study classified patients as having COVID-19 based on the earliest positive laboratory-based diagnosis or evidence of positivity in clinical notes extracted using Natural Language Processing. Patients with a positive COVID-19 test obtained on hospital days 1–5 were assumed to have been COVID-19 infected at admission. Individuals were considered to have acute COVID-19 for the first 30 days after their diagnostic test. Persons were considered to have a new episode of COVID-19 if a subsequent test was positive ≥90 days after the initial test.

Because the large sample size could result in the achievement of statistical significance despite non-meaningful differences, an *a priori* decision was made that differences <5% in the characteristics of patients with and without COVID-19 were not meaningful.

This study was approved by the VA Central Institutional Review Board.

## Results

From January 2016 through February 2020, there were 2.3 million hospitalizations among 1.05 million unique patients seen at 108 inpatient VA facilities (Table 1). Subsequently, from March 2020 through October 2022 there were 1.1 million admissions of persons without COVID-19 and 84,000 admissions of persons with COVID-19 among 620,000 and 79,000 patients, respectively; 6,320 patients had ≥1 more admission associated with a new episode of COVID-19.

**Table 1.** Patient characteristics 1/1/2016–10/31/2022

	Jan 2016–Feb 2020		Mar 2020–Oct 2022 Non-COVID-19		Mar 2020–Oct 2022 COVID-19	
Invariant data	N = 1,046,290 distinct patients		N = 619,658 distinct patients		N = 78,717 distinct patients	
Sex (N,%)						
Missing	2	0.0%	1	0.0%	1	0.0%
Female	71,510	6.8%	44,664	7.2%	4,818	6.1%
Male	974,778	93.2%	574,993	92.8%	73,898	93.9%
Race (N,%)						
American Indian or Alaska Native	7,507	0.7%	4,640	0.7%	698	0.9%
Asian	5,676	0.5%	3,867	0.6%	553	0.7%
Black or African American	223,323	21.3%	144,433	23.3%	20,562	26.1%
Two or more races	8,963	0.9%	5,500	0.9%	688	0.9%
Native Hawaiian or Other Pacific Islander	7,303	0.7%	4,628	0.7%	670	0.9%
White	753,546	72.0%	427,659	69.0%	51,273	65.1%
Missing/Uknown	39,972	3.8%	28,931	4.7%	4,273	5.4%
Ethnicity (N,%)						
Hispanic or Latino	67,081	6.4%	41,890	6.8%	6,710	8.5%
Not Hispanic or Latino	955,662	91.3%	553,351	89.3%	68,713	87.3%
Missing/unknown	23,547	2.3%	24,417	3.9%	3,294	4.2%

Admission specific data	2,264,797 admissions		1,129,736 admissions		84,452 admissions	
Age (Mean SD)	67.03	12.90	67.9	13.0	68.0	14.3
Charlson comorbidity index score (Mean, SD)	3.7	3.1	3.8	3.2	3.2	3.0
Charlson comorbidities (N, %)						
Myocardial infarction	218,455	9.6%	121,019	10.7%	6,514	7.7%
Congestive heart failure	584,933	25.8%	308,400	27.3%	18,137	21.5%
Peripheral vascular disease	452,102	20.0%	241,708	21.4%	13,355	15.8%
Cerebrovascular disease	336,619	14.9%	179,018	15.8%	11,577	13.7%
Dementia	288,116	12.7%	154,802	13.7%	12,248	14.5%
Chronic pulmonary disease	765,163	33.8%	359,121	31.8%	22,696	26.9%
Rheumatologic diseases	50,457	2.2%	27,178	2.4%	1,874	2.2%
Peptic ulcer disease	65,010	2.9%	30,008	2.7%	1,413	1.7%
Mild liver disease	136,221	6.0%	94,098	8.3%	5,158	6.1%
Diabetes without complications	313,475	13.8%	149,017	13.2%	12,370	14.6%

	Jan 2016–Feb 2020		Mar 2020–Oct 2022 Non-COVID-19		Mar 2020–Oct 2022 COVID-19	
Admission specific data	2,264,797 admissions		1,129,736 admissions		N84,452 admissions	
Diabetes with complications	655,533	28.9%	343,601	30.4%	24,116	28.6%
Hemiplegia or Paraplegia	59,020	2.6%	31,983	2.8%	2,171	2.6%
Renal disease	720,641	31.8%	394,638	34.9%	25,581	30.3%
Any malignancy	438,441	19.4%	223,176	19.8%	12,952	15.3%
Moderate-severe liver disease	186,611	8.2%	81,462	7.2%	4,278	5.1%
Metastatic solid tumor	100,677	4.4%	58,844	5.2%	2,592	3.1%
HIV or AIDS	24,213	1.1%	12,653	1.1%	820	1.0%
Drug resistance* In past year (N,%)						
MRSA colonization or infection	170,758	7.5%	57,405	5.1%	3,223	3.8%
VRE	13,124	0.6%	5,230	0.5%	281	0.3%
ESBL	12,419	0.5%	6,952	0.6%	420	0.5%
Admission type (N, %)						
Through ED	1,550,032	68.4%	842,166	74.5%	74,080	87.7%
Severity of illness (N,%)						
ICU care						
Ever	397,051	17.5%	198,976	17.6%	24,368	28.9%
Within first 24 hours	329,290	14.5%	164,739	14.6%	18,839	22.3%
Pressors						
Ever	23,995	1.1%	13,721	1.2%	2,603	3.1%
Within first 24 hours	7,511	0.3%	4,271	0.4%	317	0.4%
Admitting Service (N,%)						
Medical	1,746,054	77.1%	849,513	75.2%	68,742	81.4%
Surgery	518,743	22.9%	240,920	21.3%	8,223	9.7%
Unknown			39,303	3.5%	7,487	8.9%
Prior hospitalization within 90 days (N,%)	652,302	28.8%	319,139	28.2%	15,217	18.0%
Long-term care exposure (N,%)						
Current (within prior 7 days)	9,629	0.4%	4,008	0.4%	345	0.4%
Within prior 90 days	46,025	2.0%	15,108	1.3%	1,040	1.2%
Infectious diagnosis** (N,%)	1,144,894	50.6%	591,234	52.3%	45,273	53.6%
COVID-19 diagnosis (N,%)	108	0.0%	14,018	1.2%	74,350	88.0%
28-day mortality	109,930	4.9%	59,478	5.3%	10,313	12.2%

	Jan 2016–Feb 2020		Mar 2020–Oct 2022 Non-COVID-19		Mar 2020–Oct 2022 COVID-19	
Admission specific data	2,264,797 admissions		1,129,736 admissions		N84,452 admissions	
Length of stay						
Total days (Mean, SD)	5.4	7.3	6.2	11.2	9.2	13.3
Total days(Median, IQR)	4	2–6	4	2–6	6	3–11
ICU days (Mean, SD)	0.8	3.1	0.8	3.9	2.4	6.5
ICU days(Median, IQR)	0	0–0	0	0–0	0	0–2

All data are collected at the time of admission.

* Drug resistance was defined by report in the VA laboratory information system in the prior year indicating the detection of a methicillin-resistant *Staphylococcus aureus* (MRSA), a glycopeptide-resistant *Enterococcus spp.* (VRE) or *Enterobacteriaceae* isolate that produced an extended-spectrum beta-lactamase.

** Infection codes were determined as previously reported.^(32)^

In comparison to patients hospitalized during the pre-COVID-19 period, patients admitted without diagnoses of COVID-19 from March 2020 to October 2022 were more often admitted through the emergency department but did not differ in other demographic characteristics (sex, age, or race and ethnicity), comorbidities, severity of illness, mortality, or median length of stay. In contrast, compared with period-matched non-COVID-19 patients, patients with COVID-19 were more likely to be admitted through the emergency department or require ICU-level care, less often had prior diagnoses of congestive heart failure, peripheral vascular disease, or renal disease, less often received care on a surgical service, and were less often hospitalized in the prior 90 days. Patients with COVID-19 also had higher 28-day mortality.

**Figure 1.**
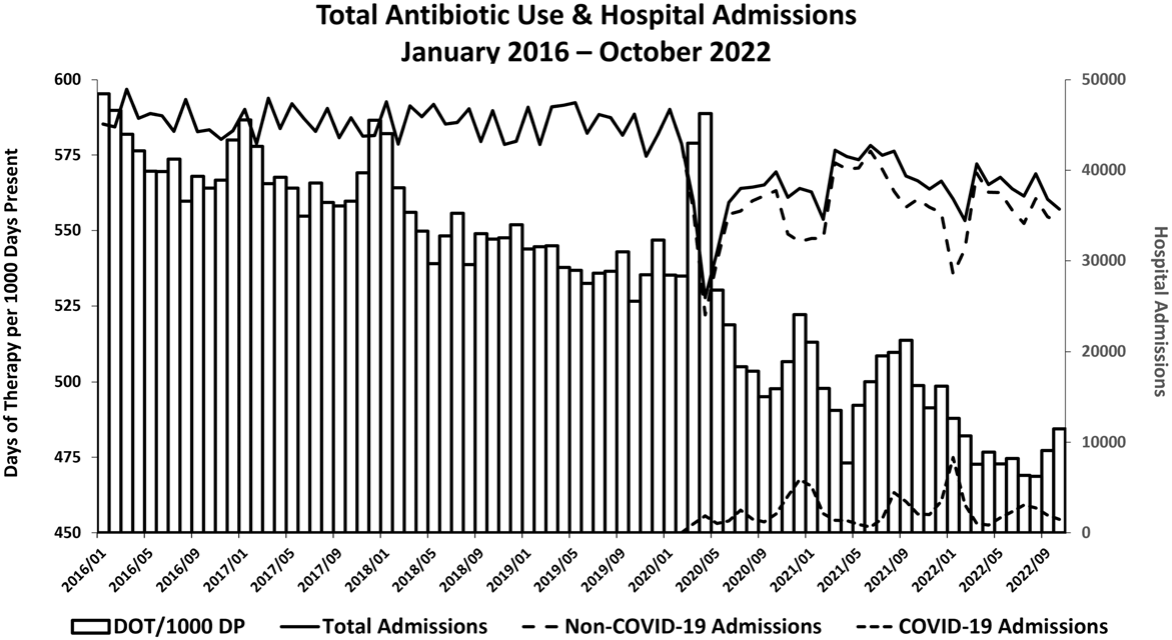
The bars represent total monthly antibiotic use, shown as Days of Therapy per 1000 Days present, for patients hospitalized on acute care and intensive care units at all included VA facilities. The solid, dashed and dotted lines represent the number of monthly admissions for all patients and patients without and with acute COVID-19, respectively.

**Figure 2.**
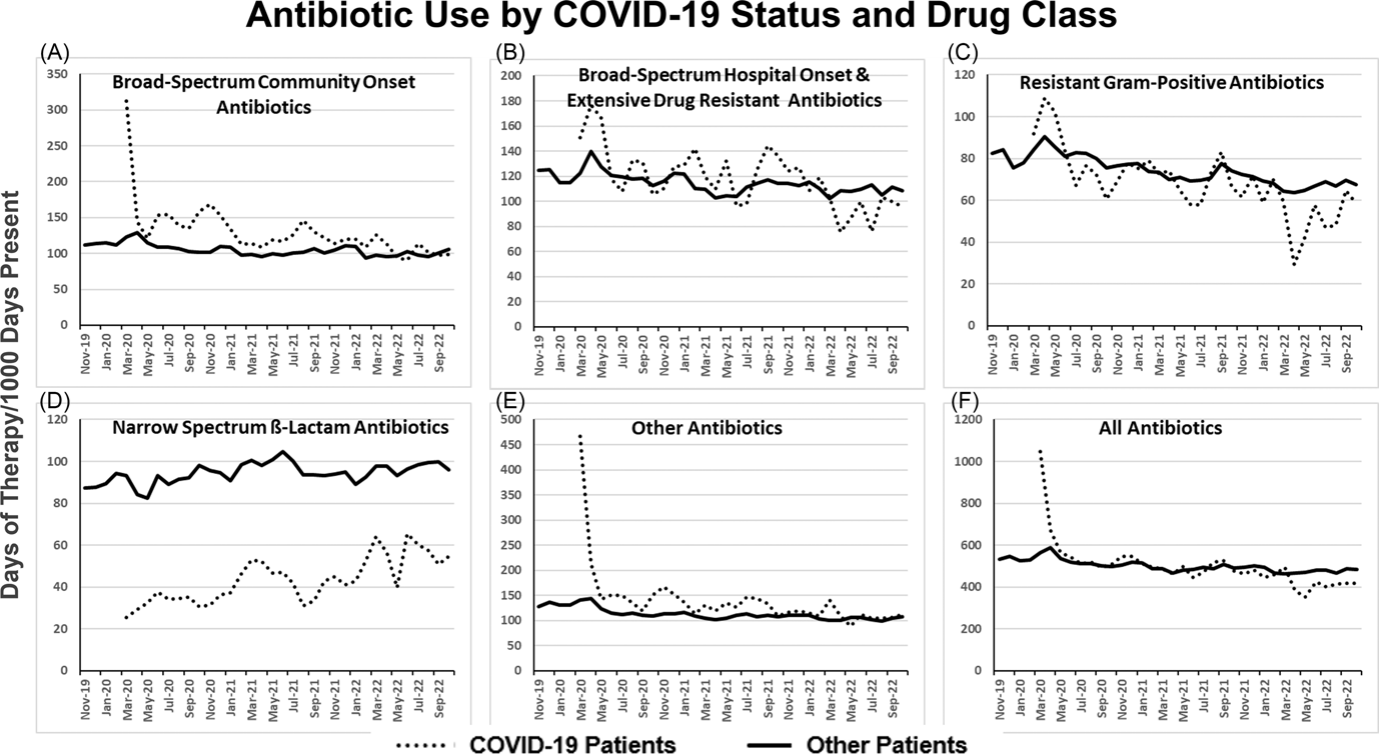
In each of the panels, the dotted and solid lines represent monthly antibiotic use by CDC NHSN antibiotic class, for hospitalized patients with and without acute COVID-19.

Mean system-wide rates of antibiotic use declined from 575 DOT/1,000 DP in 2016 to 538 DOT/1,000 DP in 2019 (Figure 1). In contrast during the first two months of the COVID-19 pandemic, the overall rate of antibiotic use for all patients increased to 588 DOT/1,000 DP; rates for patients with and without COVID-19 were 745 and 572 DOT/1,000, respectively (Figure 2, panel F). Subsequently, rates of antibiotic use quickly declined for patients both with and without COVID-19 such that antibiotic use for 2020 was less than in prior years; this downward trend continued in 2021 and 2022 (Table 2). Later surges in COVID-19 admissions during periods of peak transmission of the Delta and Omicron variants were not accompanied by substantial increases in total antibiotic use in persons with or without COVID-19 infection (Figures 1 and 2).

**Table 2.** Mean antibiotic use (DOT/1,000 DP) versus patient-level COVID-19 status

Period	Non-COVID-19	COVID-19	All
Immediately before, during and after onset of COVID-19 pandemic			
November 2019—February 2020	534	NA	534
March 2020—April 2020	572	745	588
May 2020—December 2020	512	534	515
Yearly rates			
January 2019—December 2019	NA	NA	539
January 2020—December 2020	NA	NA	528
January 2021—December 2021	491	497	492
January 2022—October 2022	475	432	470

Evaluation of CDC National Health Safety Network (NHSN) antibiotic classes showed that the use of broad-spectrum agents used for community onset infections (e.g., ceftriaxone) and antibiotics not falling within one of the CDC SAAR classes (e.g., azithromycin) was greater and the use of narrow spectrum ß-Lactam agents was less in persons with COVID-19 than in those without COVID-19 with the largest differences being in the first few months of the pandemic. There were no consistent differences in the use of Resistant Gram Positive agents or Broad-Spectrum agents for hospital-onset infections in persons with and without COVID-19 (Figure 2).

To assess whether increased antibiotic use in patients with COVID-19 was due to therapy of community onset as opposed to healthcare-associated infections, we compared rates of antibiotic use during the first ten days of hospitalization with antibiotic administration on hospital days 11–30. In the first four months of the COVID-19 pandemic, the rate of antibiotic therapy during the first ten days of hospitalization was 733 DOT/1,000 DP for patients with COVID-19 vs 548 DOT/1,000 DP for patients without COVID-19. In contrast, during hospital days 11–30, the rate of antibiotic therapy was similar in COVID-19 patients (526 vs 571 DOT/1,000 DP for patients with and without COVID-19, respectively. Patients with a second or later episode of COVID-19 had similar antibiotic use patterns to patients with their first COVID-19 episodes (data not shown).

**Figure 3.**
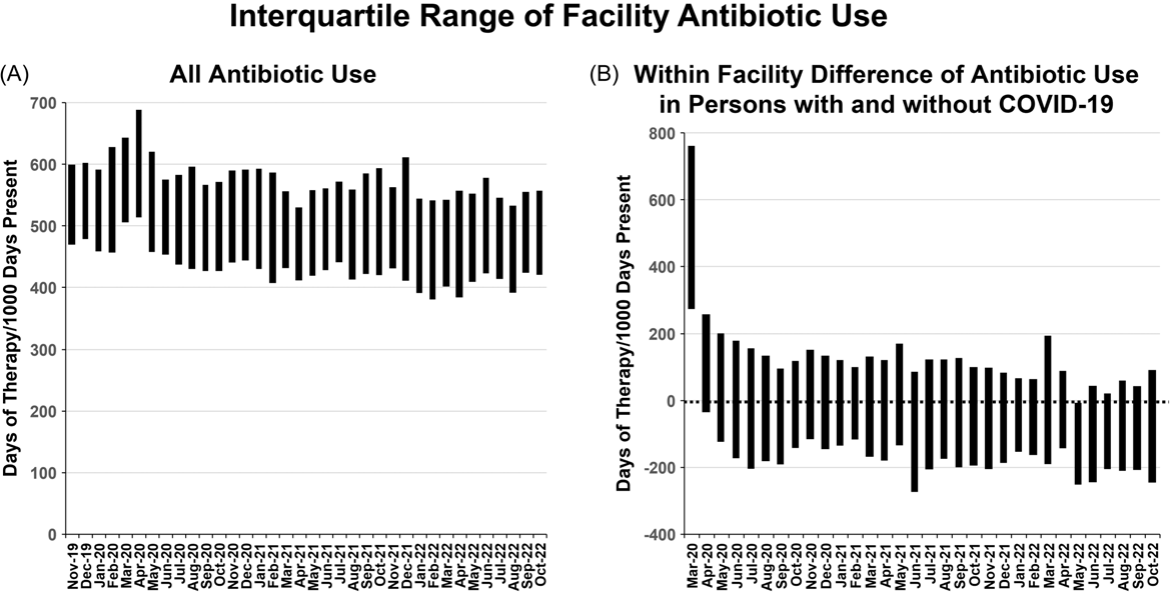
Panel A shows the interquartile range (25%–75%) of facility-level antibiotic use at all included VA facilities. Panel B shows the interquartile range of the facility-specific difference between antibiotic use in persons with and without acute COVID-19. Values greater than zero represent instances where antibiotic use was greater in persons with COVID-19 than in persons without COVID-19.

Finally, we assessed facility-level variances in antibiotic use for persons with and without COVID-19 (Table 3 and Figure 3, Panel A). In the four months prior to the pandemic, the median facility antibiotic use was 537 DOT/1,000 DP. In the first two months of the pandemic median use increased to 591 DOT/1,000 DP for all patients; rates for patients with and without COVID-19 were 739 and 574 DOT/1,000 DP. During the remaining 8 months of 2021, the median rate was 504 DOT/1,000 DP for all patients, 494 for patients with COVID-19, and 508 DOT/1,000 DP for patients without COVID-19.

**Table 3.** Median (IQR) facility-level antibiotic use versus patient-level COVID-19 status

	Non-COVID-19	COVID-19	All	COVID-19 vs non-COVID-19
	Median DOT/1,000 DP (IQR)			Median difference DOT/1,000 DP (IQR)
March 2020—April 2020	574 (494, 644)	739 (571, 946)	591 (504, 669)	201 (−37, 392)
May 2020—December 2020	508 (447, 577)	494 (352, 670)	504 (437, 585)	5.5 (−78, 124)
January 2021—December 2021	492 (428, 573)	454 (302, 626)	486 (423, 570)	−33 (−113, 57)
January 2022—October 2022	475 (410, 561)	481 (282, 547)	469 (400, 552)	−74 (−123, −1.8)

After the first two months of the pandemic, the median within facility-level rate of antibiotic use in persons with COVID-19 fell sharply and was less in patients with COVID-19 than in uninfected individuals (Table 3 and Figure 3, Panel B). However, in 2022 antibiotic use remained higher in persons with COVID-19 in 25% of facilities.

## Discussion

We previously reported that the rate of inpatient antibiotic use in VA facilities during early months of the COVID-19 pandemic was substantially higher than in previous years, thereby reversing year-to-year declines observed since 2015.^11^ We now demonstrate that rates of antibiotic use subsequently decreased substantially even as the proportion of all hospital days due to care of patients with COVID-19 increased during the Delta and Omicron surges in hospital admissions.

Increased antibiotic use during the first months of the COVID-19 pandemic was driven by high use of ceftriaxone and azithromycin among patients with COVID-19. Lesser increases were seen in the use of broad-spectrum agents for hospital-onset infections; no meaningful change occurred in the use of agents for resistant gram-positive pathogens (e.g., anti-methicillin-resistant Staphylococcus aureus (MRSA) therapy). Antibiotic use during hospital days 11–30 (i.e., for healthcare-associated infections) in patients with COVID-19 was similar to that in patients without COVID-19.

Our data expand upon the findings of others by critically evaluating the differences in antibiotic use among persons with and without COVID-19 during the pandemic. Kim *et al*. reported that among 1.1 million adults discharged with a COVID-19 diagnosis in 711 hospitals, the overall frequency of antibiotic use for non-critically ill hospitalized patients with COVID-19 decreased from 71% in 2020 to 61% in 2021.^8^ At those facilities, the median peak adjusted DOT/1,000 DP among persons with COVID-19 was over 1,300 DOT/1,000 DP and decreased by 215 DOT/1,000 DP from 2020 to 2022 with a median rate of over 600 DOT/1,000 DP in 2022.

Using data from the NHSN, O’Leary et al. demonstrated that the rate of total antibiotic use by all hospitalized patients regardless of COVID-19 status peaked in April 2020 and rapidly returned to baseline; during subsequent surges of COVID-19 smaller, transient increases in total antibiotic use were observed.^12^ However, neither of these studies directly compared rates of antibiotic therapy for persons with and without COVID-19.

Within the VA, the median facility rate of antibiotic use among persons with COVID-19 was 30% higher than in uninfected persons in March–April 2020. Subsequently, antibiotic use decreased such that total DOT/1,000 DP was 2% less in 2020 than in 2019, a trend that continued through 2022. Similar to O’Leary et al., who found a 30% decrease in inpatient days in April 2020,^12^ we found that admissions for persons without COVID-19 were 36% less in April 2020, when the peak in antibiotic use was observed, than in January 2020. Thus, it is likely that different patterns of seeking care and acuity of illness contributed to increased use of antibiotics in persons not known to have COVID-19.

This study has several limitations. First, the participating institutions were all VA facilities, which may limit generalizability. Although we assessed aggregate patient data at the facility level, we did not analyze patient-level data and or the appropriateness, indication, or duration of therapy; furthermore, we did not correct for differences in patient characteristics at the individual or facility level. We also may not have captured all initial SARS-CoV-2 diagnose for patients had initial positive SARS-CoV-2 tests at non-VA locations.

Many factors, including guidelines recommending against routine antibiotic use,^13,14^ increased awareness of low rates of coinfection,^6^ codification of risk factors for bacterial infection,^7,15^ increased willingness to obtain lower respiratory tract specimens for culture with better infection control practices, and changes in disease severity due to increased levels of immunity and changes in viral virulence,^16^ are likely to have contributed to decreased antibiotic utilization after the initial months of the COVID-19 pandemic. Nonetheless, our findings point to the resiliency of antimicrobial stewardship within VA, which has been a product of several factors: the establishment of an Antimicrobial Stewardship Task Force in 2011,^17^ organizational directives establishing and updating recommendations for antimicrobial stewardship program staffing, system-wide medication use evaluations that identify opportunities for improvements in antimicrobial use,^18–20^ development of regional antimicrobial stewardship collaboratives, and strong implementation science engagement and utilization of informatics-based tools.^21^

However, despite system-wide quality improvement initiatives, policies and staffing guidelines, variances in antibiotic use persist in the VA. Reported contributors to variation within the VA to include differing levels of staffing for stewardship programs,^22,23^ availability of infectious diseases specialists, variations in “antibiotic prescribing etiquette,”^24,25^ and implementation of nationally recommended programs.^26–28^ Further adding to the variations in stewardship practices during the period in question was the impact of COVID-19 on infection control and antimicrobial stewardship programs.^29,30^

In summary, the rapid normalization of antibiotic use in persons with COVID-19 over the course of the pandemic and the continued downward trend in antibiotic use throughout the VA is reassuring, especially since many antimicrobial stewards’ usual activities were co-opted by pandemic-related tasks.^29,30^ However, substantial variations in antimicrobial use persisted across institutions. While the most recent rates of antibiotic use were substantially lower in persons with COVID-19 than in non-COVID-19 patients, ongoing interfacility variance in antibiotic use for persons with COVID-19 suggests that despite guidelines to the contrary and the low prevalence of community-onset infections in persons with COVID-19^6,13,14^ substantial opportunities remain for improved antibiotic stewardship in some facilities. Further research will allow for ongoing refinement of antimicrobial stewardship goals and priorities within VA over the next several years.^31^

## Supporting information

Goetz et al. supplementary material Goetz et al. supplementary material

## Supplementary material

For supplementary material accompanying this paper visit http://doi.org/10.1017/ash.2024.352. click here to view supplementary material
