# Supplementary material for: Antimicrobial use before and during COVID-19: data from 108 Veterans Affairs medical centers
Source: Antimicrob Steward Healthc Epidemiol. 2024 Aug 7;4(1):e109. doi: 10.1017/ash.2024.352 (PMC11736461; doi:10.1017/ash.2024.352)
Supplement: Goetz et al. supplementary material [file S2732494X24003528sup001.docx]

**Supplementary Table 1**

**CDC-defined adult standardized antimicrobial administration ratio (SAAR) antibiotic categories (21)**

**Adult Broad spectrum antibacterial agents predominantly used for hospital-onset infections**

Amikacin (IV only)

Aztreonam (IV only)

Cefepime

Ceftazidime

Doripenem

Gentamicin (IV only)

Imipenem/cilastatin

Meropenem

Piperacillin/tazobactam

Tobramycin (IV only)

**Adult** **Broad spectrum antibacterial agents predominantly used for community-acquired infections**

Cefaclor

Cefdinir

Cefixime

Cefotaxime

Cefpodoxime

Cefprozil

Ceftriaxone

Ciprofloxacin

Cefuroxime

Ertapenem

Gemifloxacin

Levofloxacin

Moxifloxacin

**Adult Antibacterial agents predominantly used for resistant Gram-positive infections (e.g., MRSA)**

Ceftaroline

Dalbavancin

Daptomycin

Linezolid

Oritavancin

Quinupristin/dalfopristin

Tedizolid

Telavancin

Vancomycin (IV only)

**Adult Narrow spectrum beta-lactam agents**

Amoxicillin

Amoxicillin/clavulanate

Ampicillin

Ampicillin/sulbactam

Cefadroxil

Cefazolin

Cefotetan

Cefoxitin

Cephalexin

Dicloxacillin

Nafcillin

Oxacillin

Penicillin G

Penicillin V

**Adult Antibacterial agents predominantly used for extensively antibiotic resistant bacteria**

Ceftazidime/avibactam

Ceftolozane/tazobactam

Colistimethate (IV only)

Polymyxin B (IV only)

Tigecycline
